# Aging Modulates the Resting Brain after a Memory Task: A Validation Study from Multivariate Models

**DOI:** 10.3390/e21040411

**Published:** 2019-04-17

**Authors:** Garazi Artola, Erik Isusquiza, Ane Errarte, Maitane Barrenechea, Ane Alberdi, María Hernández-Lorca, Elena Solesio-Jofre

**Affiliations:** 1 Biomedical Engineering Department, Mondragon Unibertsitatea, 20500 Mondragón, Gipuzkoa, Spain; 2 Departamento de Psicología Biológica y de la salud, Facultad de Psicología, Universidad Autónoma de Madrid, 28049 Madrid, Spain

**Keywords:** electroencephalogram (EEG), resting state, time-frequency analysis, machine learning

## Abstract

Recent work has demonstrated that aging modulates the resting brain. However, the study of these modulations after cognitive practice, resulting from a memory task, has been scarce. This work aims at examining age-related changes in the functional reorganization of the resting brain after cognitive training, namely, neuroplasticity, by means of the most innovative tools for data analysis. To this end, electroencephalographic activity was recorded in 34 young and 38 older participants. Different methods for data analyses, including frequency, time-frequency and machine learning-based prediction models were conducted. Results showed reductions in Alpha power in old compared to young adults in electrodes placed over posterior and anterior areas of the brain. Moreover, young participants showed Alpha power increases after task performance, while their older counterparts exhibited a more invariant pattern of results. These results were significant in the 140–160 s time window in electrodes placed over anterior regions of the brain. Machine learning analyses were able to accurately classify participants by age, but failed to predict whether resting state scans took place before or after the memory task. These findings greatly contribute to the development of multivariate tools for electroencephalogram (EEG) data analysis and improve our understanding of age-related changes in the functional reorganization of the resting brain.

## 1. Introduction

Aging is one of the primary health concerns in the entire world. Hence, understanding the aging process as well as its neural underpinnings is crucial to improve seniors’ functioning in daily life. The major part of the studies on healthy aging have approached it as a decadent period of decline in both physical and cognitive domains [1,2,3,4]. However, older individuals also exhibit a strikingly well-preserved ability to process emotions [5].

Under this perspective of positive aging, recent research has also demonstrated that older individuals’ capacity to learn new information is associated with a functional and structural reorganization of the brain, so-called neuroplasticity. In this regard, most of the studies have explored neuroplasticity in relation to cognitive and motor improvement [6,7,8,9]. However, the benefit of utilizing spared emotional abilities in order to help mitigate the memory decline that occurs with age has not been previously explored.

This is precisely the main objective of the present study, belonging to the project MEMOTION. This project aims to analyse age-related changes in neuroplasticity, particularly in neural networks underlying emotional and memory processes using a training program, focused on the enhancement of preserved emotions in order to improve memory deficits. This paper presents a first step towards this major goal, validating the resting state approach to test neuroplasticity. For this purpose, we focus on the analysis of the resting brain before and after a memory task performed prior to the training program.

For this study, we used electroencephalography (EEG), which is an electrophysiological monitoring system to record electrical activity of the brain, specifically from the post-synaptic pyramidal neurons [10]. It is typically non-invasive and acquired by means of electrodes that are placed along the scalp. These electrodes measure voltage variations resulting from ionic currents within the neurons of the brain. The excellent temporal resolution of the EEG is ideal to assess rapid cognitive events that occur in the order of milliseconds. EEG signal analysis generally focuses on either event-related potentials (ERP), where the potential variations that are time-locked to an event-like stimulus are investigated, or on the spectral content of the signal. The latter analyses focus on the type of neural oscillations or brain waves that can be observed in the frequency domain [11]. The frequencies of the brain signals are classified in different waves depending on the frequency band and the state where they appear: Delta = 0.5–4 Hz, Theta = 4–8 Hz, Alpha = 8–12 Hz, Beta = 12–30 Hz and Gamma = 30–48 Hz. As opposed to the ERPs, this type of neural oscillations can be also analysed in a resting-state. Resting-state EEG (rs-EEG) refers to the recording of the brain’s electrical activity when a subject is not performing an explicit task. Recent research has devoted increasing attention to the brain at rest [11]. In this regard, rs-EEG has been proved as a reliable indicator of functional reorganization of brain networks, supporting a wide range of perceptual and goal-directed tasks [12,13,14]. Converging evidence regarding the functional relevance of spontaneous brain activity has revealed prominent differences not only in patients with brain disorders [15,16] but also in healthy ageing [2,17]. However, not a single study to date has examined age-related changes in neuroplasticity after a training program based on positive emotions by means of rs-EEG.

**Figure 1:**
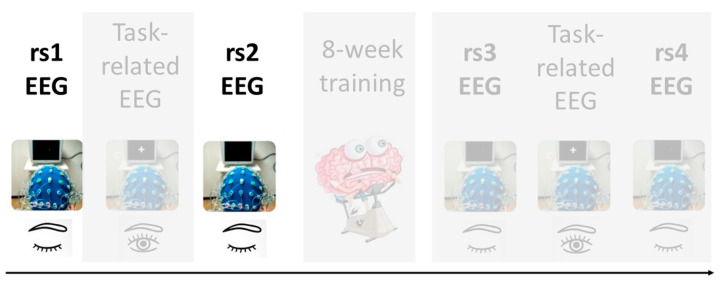
Data acquisition protocol for the MEMOTION project. The present study focuses on the highlighted resting-state electroencephalogram (rs-EEG) prior to the eight-week training program.

Our study design consists on a series of task-related and rs-EEG scans recorded before and after an eight-week training program in positive emotions to later examine the neuronal changes that occur in young and older individuals as a result of this training. This article describes the analysis of rs-EEG before such a training program, particularly, of two resting state scans that occur before and after the performance of a memory task (see Figure 1 for further clarification). Importantly, we have used very innovative approaches for EEG data analysis, including different time-frequency methods and machine learning models.

EEG has generally been studied using time-frequency analysis. In previous studies, the Morlet wavelet transform (MWT) has been used in order to find statistically significant differences in the Alpha band using a time window length of 30 s [18]. The work presented in this article is an extension of this research. In this respect, two different time-frequency analyses and a variety of time window lengths have been tested. The rationale for this is twofold: on the one hand, different time-frequency analysis techniques are performed with the aim of increasing the validity of the results. The performance of these time-frequency analysis methods depends strongly on the selected parameters, therefore, a qualitative analysis between two time-frequency analysis techniques has been carried out. On the other hand, reducing the length of the analysis windows provides a better time estimate of the instant at which the changes in the rs-EEG occur. Therefore, the MWT and the short-term Fourier transform (STFT) have been used to obtain the time-frequency maps. Additionally, six different time window (TW) lengths (5 s, 10 s, 15 s, 20 s, 25 s and 30 s) have been considered.

Furthermore, with the aim of classifying both age groups and resting state instances, machine learning techniques have been employed. Machine learning is a subdiscipline of artificial intelligence (AI) whose principal objective is to generate algorithms able to learn and/or adapt their own structure, based on the observed data [19,20]. In the case of high-dimensional or multimodal data, as it is the case in most biomedical applications, this technique facilitates the analysis. On the one hand, it can be used to discover both linear and non-linear data patterns that classic statistical methods cannot detect. On the other hand, machine learning algorithms deal better with multivariate relationships, bringing into light patterns that may otherwise go unnoticed. For this purpose, there are several well-known methods, classified into regression (e.g., linear regression, support vector regression or k nearest neighbours (kNN)) or classification methods (e.g., support vector machines (SVM), AdaBoost, multilayer perceptron or random forest (RF)). The formers predict continuous variables, whereas the latter determine categorical class labels. Further information about these techniques can be found in [19,20]. In this study, decision tree (DT), kNN, linear discriminant analysis (LDA), RF and SVM models have been trained and tested.

Previous research has demonstrated that the use of machine learning and deep learning techniques for the analysis of a wide variety of health-related data can be useful in detecting underlying patterns otherwise difficult to identify. Some examples are Google’s AI system that predicts cardiovascular risk factors from retinal fundus photographs via deep learning [21]. In this study they predicted cardiovascular risk factors not previously thought to be present or quantifiable in retinal images, such as age, gender, smoking status, systolic blood pressure and major adverse cardiac events. In [22] the authors used machine learning techniques to detect retinal lesions such as haemorrhages and microaneurysms, while the work presented in [23] introduced an automated diabetic retinopathy severity grading system based on retinal fundus images. In addition, in [24], machine learning techniques were used for the prediction of glaucoma risk probability. Finally, EEG-based machine learning models have been proved to be useful in stress detection [25] and in distinguishing between healthy elderly and patients with Alzheimer’s disease or mild cognitive impairment [26,27].

In this regard, there has been an increased interest in building age-predictive models based on brain-related measures (signal or image) in the recent years. Cole et al., 2015 [28] predicted the brain age of different subjects after a traumatic brain injury (TBI). To this end, a normal ageing predictive model developed by machine learning was used to analyse images obtained by magnetic resonance imaging (MRI). Results suggested that TBI can accelerate the brain atrophy: the model was able to predict the age of the healthy subjects, whereas it provided higher age values for patients who suffered a TBI. Following the same trend, Franke et al. [29] introduced a framework for automatically estimating the age of healthy subjects from their T1-weighted MRI scans using a kernel method for regression in their study. Two years later, another study [30] was conducted using the same framework, which provided a validated reference curve for structural brain maturation during childhood and adolescence. Additionally, Camino-Pontes et al., 2018 [31] analysed how the values of redundant and synergetic interactions in dynamical networks are distributed across brain areas and along the lifespan of a subject using functional magnetic imaging data. They found a robust balance between redundant and synergetic interactions across brain areas and along lifespan, demonstrating compensatory informational mechanisms in brain networks.

The goal of this study was to assess age-related changes in the resting brain before and after the performance of a cognitive task in order to test neuroplasticity and validate the resting state approach as a predicting tool for cognitive differences between young and older adults. Based on previous studies [2,18], we expected age-related differences in resting state neuroplasticity following task performance. To test this hypothesis, we introduced very innovative approaches for EEG data analysis, including different time-frequency methods and machine learning models in order to validate these methods for data analysis in the context of aging and neuroplasticity. Specifically, we first used frequency and different time-frequency techniques to identify the frequency bands, time windows and channels of interest where aging differences in neuroplasticity occurred. Additionally, we were able to validate the different time-frequency methods employed. Then, we built prediction models in order to answer two specific research questions: (1) “*Are EEG oscillations able to predict the age of the participant under assessment?*” (2) “*Are EEG oscillations able to distinguish between resting state scans before and after the cognitive task?*” 

## 2. Materials and Methods

This section describes how the EEG recordings and data acquisition have been carried out. Additionally, the signal pre-processing and processing steps are described, along with the implemented data analysis and machine learning techniques.

### 2.1. Subjects

The study included 34 young (22.15 ± 1.20 years, age range: 18–26 years) and 38 older (66.81 ± 3.45 years, age range: 60–75 years) participants. All of them had normal or corrected-to-normal vision, and were right-handed according to the Edinburgh Handedness Inventory (Oldfield, 1971). They were naive with respect to the experimental paradigm. None of the participants reported a history of neurological, psychiatric, or vascular disease. Older participants were screened for cognitive impairments with the Montreal Cognitive Assessment test (MoCA) using the standard cutoff score of 26 (Nasreddine et al., 2005). All participants obtained a score within normal limits (≥26, mean = 27.82, standard deviation (SD) = 1.45, range = 26–29). Informed consent was obtained before testing and participants were financially compensated for participation. The experiment was approved by the local ethics committee for biomedical research of University Autónoma of Madrid (Spain), and was performed in accordance with the Declaration of Helsinki (1964).

### 2.2. Experimental Setup

EEG recordings occurred twice: before (pre-test session) and after (post-test session) a training program of 8 weeks of duration. Both EEG sessions had identical design with a total duration of 1.5 h each. Therefore, the overall experimental procedure was as follows: the pre-test session included a rest scan (rs1), followed by a task-related scan (tr1), after which another rest scan (rs2) was obtained. The pre-test session was followed by a training program in positive emotions composed of 10 sessions of 2 h each during an 8-week period. Following completion of this training program, the post-test session included a rest scan (rs3), followed by a task-related scan (tr2), and subsequently another rest scan (rs4). See Figure 1 for data acquisition protocol. The current work focuses on the analysis of the rs-EEG scans before the training program (rs1 and rs2). All rest scans had the same protocol and lasted 5 min, in which participants were instructed to keep their eyes open and to fixate a target point. The task-related scans were recorded during a memory task which was distributed in a study-test design. During the study stage, 150 pictures were shown, and participants were just instructed to watch images as if they were watching tv at home. During the test stage, 300 pictures were shown (150 pictures were included in the study stage and the remaining 150 were new pictures), and participants were required to respond with a button press to indicate if they had seen or not each picture during the study stage.

### 2.3. Electroencephalogram (EEG) Data Acquisition

Data were recorded using BioSemi bioactive electrode caps with 128 EEG channels and five external electrodes, one placed at the tip of the nose (as a potential off-line reference electrode) and four as horizontal and vertical EOG channels. Offsets of the active electrodes were kept below 25–30 millivolts. Data were digitalized at a sampling rate of 1024  Hz and low-pass filtered online at 100 Hz.

### 2.4. Signal Pre-Processing

EEG data analysis was conducted using the EEGLab toolbox [32] run under Matlab (R2015a, MathWorks). The EEG signal was firstly downsampled to 256 Hz and off-line filtered between 1 and 45  Hz, in accordance with previous research considering these cutting frequencies as frequencies of interest in resting state studies [33,34,35,36,37,38,39]. Noisy channels were detected and linearly interpolated based on the activity of neighbouring electrodes. Then, the signal was referenced to a common average reference. In addition, trials showing eye blinks or other artifacts such as cable movement or muscular artifacts were identified and removed by means of independent component analysis (ICA). Subsequently, bad channels were interpolated again using the signals of the adjacent denoised channels. Finally, the central segment of the 5 min EEG recordings were kept for further processing and analysis, as the initial and final intervals of the EEG recording showed artifacts related to subject adjustment and other environmental disturbances. More specifically, the first 5 s of each recording were discarded, saving only the following 256 s (4 min and 16 s) of each EEG recording.

### 2.5. Frequency Analysis

Subsequently, we calculated the mean power of each scan session (rs1, rs2), frequency band (Delta = 0.5–4 Hz; Theta = 4–8 Hz; Alpha = 8–12 Hz; Beta = 12–30 Hz; Gamma = 30–48 Hz) and channel (128 in total) for young and older participants.

### 2.6. Time-Frequency Analysis

Given the nature of the current EEG study, further analysis into the Alpha band (where the neural activity concentrates during resting state) was conducted. When analysing biosignals it is often interesting to see how the frequency content of the signal changes over time. These changes can be noticed by using time-frequency analysis techniques, such as the STFT, or wavelet-based approaches such as the MWT.

The STFT, also termed spectrogram, slices the analysed waveform into a number of short segments, possibly overlapping. Then, it applies a window function to each segment, and subsequently the Fourier transform (FT) is applied to each one of the windowed segments. The main shortcoming of this method stems from the need to select an appropriate window length. If the window length is short, the STFT can track changes that occur very close in the time domain and, therefore, the time resolution is very good. However, due to the fact that the data segments where the FT is applied are short, the frequency resolution of the STFT is compromised. The opposite happens when a long window is selected: a good frequency resolution is achieved at the expense of time resolution.

The problem of selecting an appropriate window length is partially resolved by using a wavelet as the basis function in the decomposition of the signal into the frequency domain. When a wavelet is used to create a time-frequency map, the reference wavelet (also termed the mother wavelet) is scaled in order to account for different time and frequency scales. This provides the wavelet with a built-in time-frequency trade-off, where longer wavelets are used to analyse the low-frequency content of the signal, whereas shorter wavelets that can attain a better time resolution are used in the higher part of the spectrum. The wavelet that has been used in this study, the MWT, is a complex wavelet comprising real and imaginary sinusoidal oscillations, which is convolved with a Gaussian envelope so that the wavelet magnitude is largest at its centre and tapered toward its edges [40,41].

The parameters used in this study for the STFT have been a window length of 256 samples, corresponding to a 1 s window, with no overlapping. As for the MWT, a wavelet factor of 7 and 256 frequency bins in the 0.5–48 Hz frequency range have been used [42]. Both time-frequency analysis methods have been implemented and compared qualitatively for different time windows: 5 s, 10 s, 15 s, 20 s, 25 s and 30 s.

### 2.7. Statistical Analysis

EEG data obtained from frequency analyses were subjected to a 2 × 2 × 5 × 128 (age × scan session × frequency band × channel) repeated measures ANOVA, each. Here, age (young, older) was the between-subject factor. Scan session (rs1, rs2), frequency band (Delta, Theta, Alpha, Beta and Gamma) and channel (1…128) were the within-subject factors. False discovery rate (FDR) [43] correction for multiple comparisons was applied and those results surviving the correction with an adjusted level of significance at *p* < 0.05 were considered as significant effects.

Then, Alpha band EEG data obtained from time-frequency analyses of the 42 channels that showed significant results in the previous step were subjected to a 2 × 2 × nº_TW_ × 42 (age × scan session × time-window × channel) repeated measures analysis of variance (ANOVA), each. Here, again, age (young, older) was the between-subject factor. Scan session (rs1, rs2), time-window (1...nº_TW_) and channel (1…42) were the within-subject factors. FDR correction for multiple comparisons was applied with an adjusted level of significance at *p* < 0.05.

Finally, we further analysed the identified significant differences to look for overall patterns by computing the average values of the observations per group (young, old) and scan session (rs1, rs2) in both frequency and time-frequency-domains.

### 2.8. Machine Learning-Based Analysis

After the statistical analysis, we built machine learning-based prediction models in order to evaluate the classification ability of the EEG waves for specific subject groups (young, old) and conditions (rs1, rs2). These models aim to answer the research questions posed in Section 1: (1) “Do EEG waves’ frequency content predict the age-group of the subject that is being monitored?” (2) “Do EEG waves’ frequency content distinguish between pre- and post-task situations?”

To answer these questions, we used the frequency-domain pre-processed dataset with the aim of identifying the neural rhythm, channel or scan session with the highest classification ability. We built prediction models based on LDA, kNN, RF, radial kernel-based SVM and DT. We used the implementations of the “caret” library available in R for the algorithms.

For the first research question, we used age (young, older) as the response variable, whereas power of the Alpha, Beta, Gamma, Theta and Delta frequency bands, scan session (rs1, rs2) and channels (128) were the predictors. For the second research question, scan session (rs1, rs2) was considered as the response variable, whereas age (young, older), power of the frequency bands (Alpha, Beta, Gamma, Theta and Delta) and channels (128) were the predictors. The reason why all frequency bands have been used in the models, rather than focusing on Alpha (which is the one that has shown significant results in the statistical analysis) is twofold. On the one hand, machine learning techniques may find linear and non-linear relationships between variables that may go unnoticed when using classical statistical techniques. On the other hand, they can deal better with multivariate relationships, hence the interest in including all the potentially explanatory variables of our research questions in the models.

All models were built and evaluated following a leave-one-subject-out cross-validation (LOSOCV) approach. Having available multiple data-instances for each subject, the data are not-independent and, thus, the use of the most common evaluation techniques such as the 10-fold cross validation would result in overoptimistic classification accuracies. Therefore, more specific validation techniques must be used. LOSOCV, consists on using data from all subjects except for one to train the models, and to evaluate the prediction accuracy on the remaining subject. This process is then repeated until all subjects have been used as testing data. Following this approach, we evaluated the accuracy, sensitivity, specificity and F-score of the machine learning models. The combination of these metrics offers an excellent overview of both the models’ overall performance and the capability of correctly detecting the two target classes. In addition, we also analysed the variability of the models’ accuracy across folds (i.e., across subjects) in order to evaluate the stability of the results for different subjects.

Finally, we aimed at evaluating the contribution of each variable to each one of the research questions. For this goal, we computed the relative feature importance of the predictors using a RF model with the Mean Decrease in Accuracy criteria [44]. This technique calculates the accuracy of the trees that build the RF for the out-of-the bag sample of each tree. Then, it permutes the variables one after the other and measures back the accuracy to calculate the decrease without the variable under analysis. Results obtained across trees are averaged to obtain relative feature importance. We visualized the top 10 features as ranked by the algorithm, together with their relative importance for the two research questions.

## 3. Results

This section summarizes the results obtained through statistical analysis of frequency band power and time- frequency power data, along with the machine learning analysis results.

### 3.1. Frequency Analysis Results

Age-related changes in mean power during scan sessions for each frequency band and channel were assessed with a 2 × 2 × 5 × 128 (age × scan session × frequency band × channel) repeated measures ANOVA.

A significant age × scan session interaction effect was found in 42 of the EEG channels for the Alpha band, indicating that, change from pre-task to post-task sessions differed between young and old subjects. The detailed results (F values and *p*-values) obtained for the significant age × scan interaction terms can be seen in Table 1.

**Figure 2:**
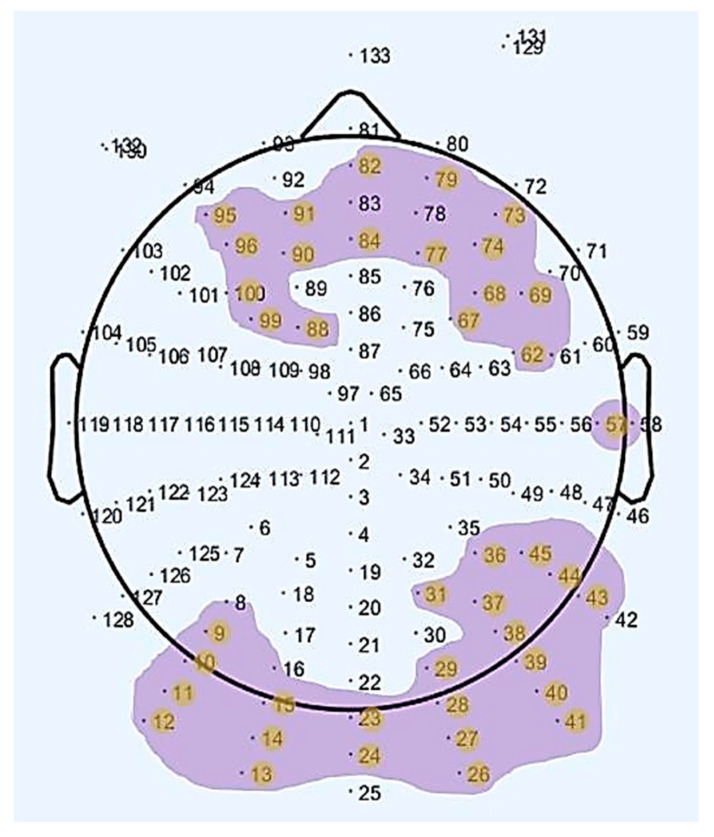
Map of the scalp showing the channels with a statistically significant difference regarding the interaction between age group and rs-EEG scan in the Alpha waveform.

The electrodes where statistically significant results were obtained, as seen in Table 1, are plotted in Figure 2. As can be observed in the aforementioned figure, we obtained statistically significant differences in electrodes placed over frontal, prefrontal, occipital and posterior areas of the brain for the two age groups (old, young) between resting-states (rs1, rs2).

**Figure 3:**
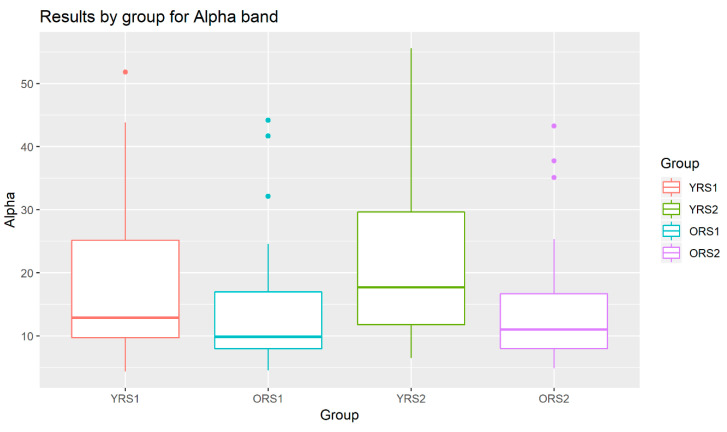
Boxplots of the Alpha band power results in channel 27 for young adults in rs1 (YRS1), older adults in rs1 (ORS1), young adults in rs2 (YRS2) and older adults in rs2 (ORS2).

The analysis of the grand mean values showed that although both age groups suffered a positive increase in the Alpha band power of all channels, this change from pre- to post-memory task was more evident in young adults than in old adults. Moreover, the values for the young group were in all cases higher than for the elderly. An example of these patterns can be observed in Figure 3, where results for the Alpha waveform in the 27th channel are shown.

### 3.2. Time-Frequency Analysis Results

Age-related changes in mean alpha power during scan sessions for each time-window and channel were assessed with a 2 × 2 × nº_TW_ × 42 (age × scan session × time-window × channel) repeated measures ANOVA. Significant age × scan session interaction effects were found in 23 of the EEG channels under analysis distributed over several time-windows for the alpha band using the MWT time-frequency analysis technique, and in *37* channels using the STFT technique. This indicates that, changes from first to second resting state conditions differed between young and older subjects in certain time-windows in these specific channels. The similarity in the results obtained using both time-frequency techniques reaffirms the results.

To be more specific, all time-frequency methods have shown significant results around 140–160 s, with a higher incidence in channels over frontal (channels 62–100) than over occipital (channels 9–45) regions. When smaller window lengths are studied, such as 5 and 10 s, the same pattern has been observed before applying the FDR. However, once the correction has been applied no statistically significant results have been obtained, due to the higher number of comparisons that are made. Around 80 s, statistically significant results are found in channels over frontal and occipital areas equally for 30 s windows in the two studied time-frequency analysis techniques. When smaller windows are considered, such as 20 s and 25 s windows, statistically significant results are obtained also in the vicinity of 80 s. However, these results are not so prominent as they are captured in two consecutive windows. Finally, statistically significant differences can be observed at the beginning of the selected 256 s interval in the results obtained by means of the STFT.

**Figure 4:**
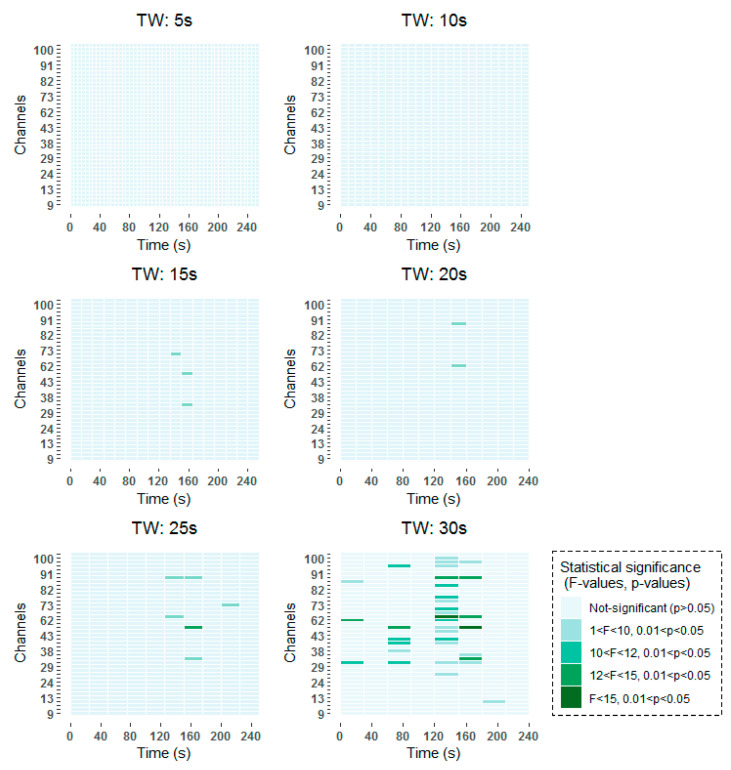
Significant time windows after FDR correction for the Morlet wavelet transform (MWT) time-frequency maps in the Alpha band using 5 s, 10 s, 15 s, 20 s, 25 s and 30 s windows.

**Figure 5:**
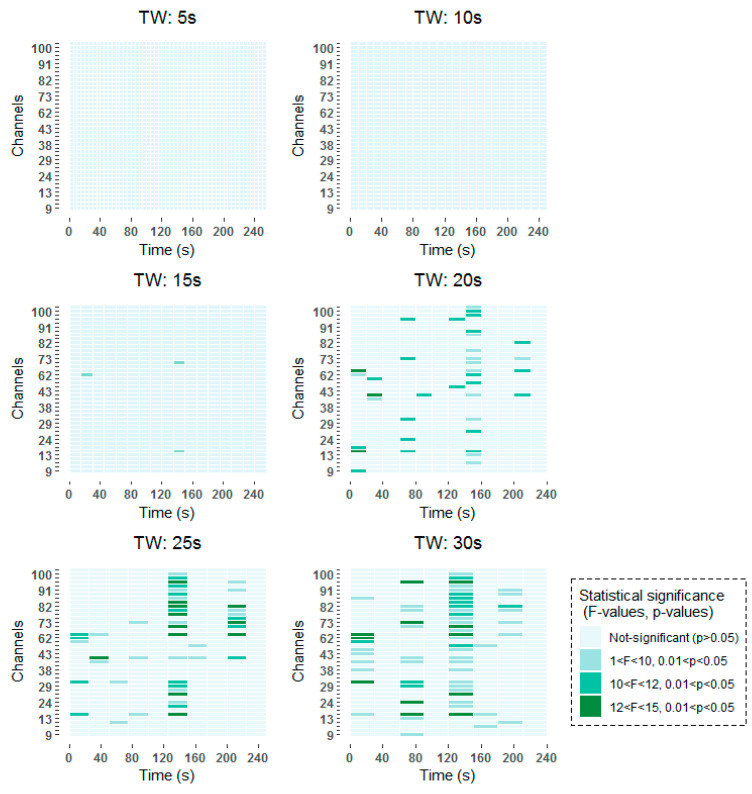
Significant time windows after FDR correction for the short-term Fourier transform (STFT) time-frequency maps in the Alpha band using 5 s, 10 s, 15 s, 20 s, 25 s and 30 s windows.

The detailed results of the time-frequency analysis in the Alpha band after FDR correction at a significance level of *p* < 0.05 are shown in Figure 4 and Figure 5.

**Figure 6:**
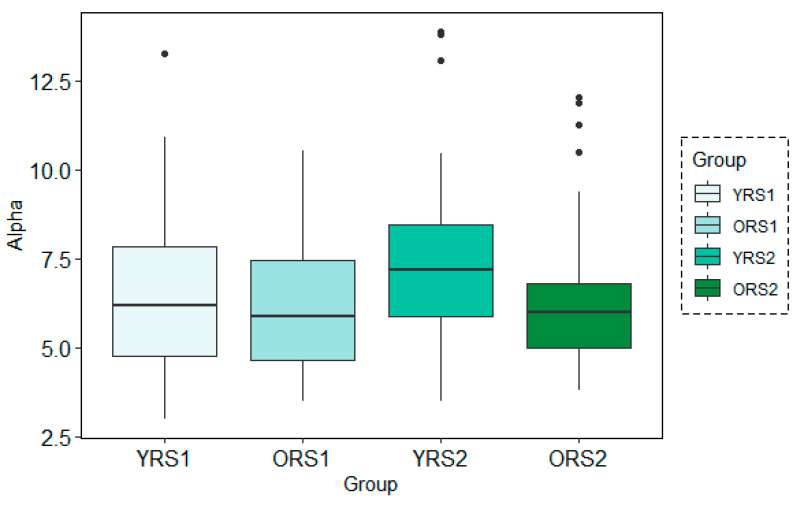
Boxplots of the Alpha band power results for each group (young adults in rs1 (YRS1), older adults in rs1 (ORS1), young adults in rs2 (YRS2) and older adults in rs2 (ORS2)) in the channel 67 at time-period 120–150 s using the MWT technique with the 30s time-window configuration.

**Figure 7:**
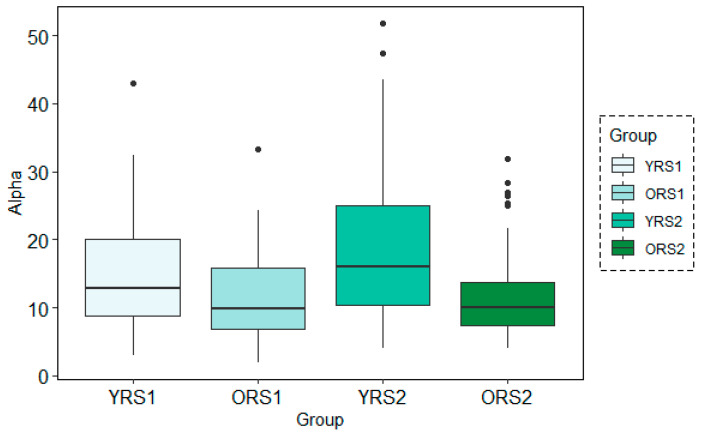
Boxplots of the Alpha band power results for each group (young adults in rs1 (YRS1), older adults in rs1 (ORS1), young adults in rs2 (YRS2) and older adults in rs2 (ORS2)) in the channel 67 at time-period 120–150s using the STFT technique with the 30s time-window configuration.

The analysis of the grand mean values showed again the same tendency as in the frequency-domain for both analysis techniques: MWT and STFT. Using both methods, there was a positive increase in the alpha band power from pre- to post-memory task resting state scans more evident in young adults than in older adults. Moreover, power values for young participants were in all cases higher than for the elderly. Figure 6 and Figure 7 depict an example of this pattern of results for channel 26 at the time-period 120–150 s analysis using a temporal window of 30 s with MWT and STFT, respectively.

### 3.3. Machine Learning Analysis Results

**Figure 8:**
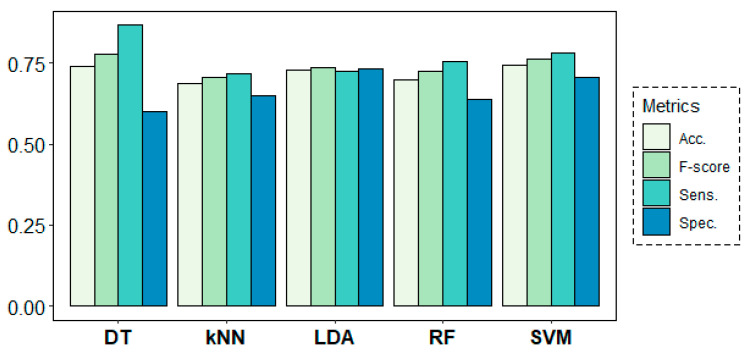
Classification results for the age-group target variable using a leave-one-subject-out cross-validation (LOSOCV) approach.

Figure 8 shows the results of the classification models for the age-group prediction using EEG data from all frequency bands of all channels in both rs1 and rs2. All models yielded accuracy results above 68% and F-score values over 0.70. Best results were obtained by the DT algorithm with 74% of accuracy and an F-score of 0.78, despite showing a weak 0.62 value in terms of specificity. These results were closely followed by the SVM algorithm, which achieved much more stable results of 74% of accuracy and 0.78, 0.70 and 0.76 values for sensitivity, specificity and F-score, respectively.

**Figure 9:**
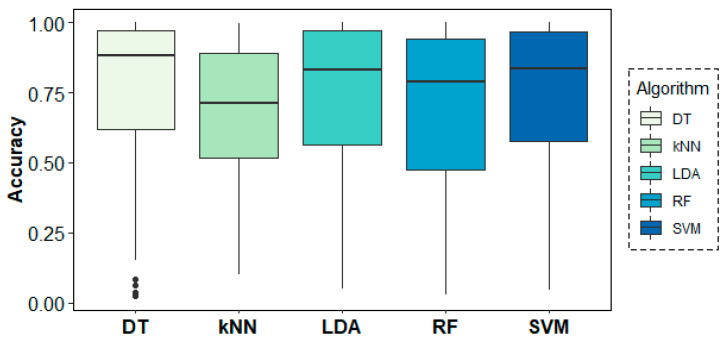
Classification accuracy results across folds (subjects) for the age-group target variable.

Figure 9 shows the behaviour of the models across folds (i.e., across subjects), where over-chance prediction accuracy can be observed for the age-group target variable for almost all subjects. The DT algorithm shows the highest accuracy and most stable results across folds, followed by SVM and LDA.

**Figure 10:**
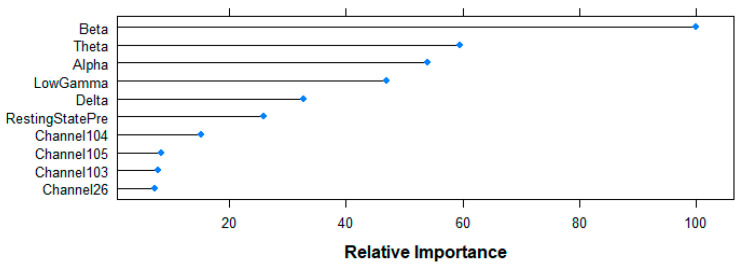
Relative importance of the top 10 features for the age-group prediction task following random forest (RF)-based Mean Decrease in Accuracy criteria.

Figure 10 shows the top-10 ranked variables by the RF algorithm for the prediction of the age-group following the Mean Decrease in Accuracy criteria. Beta-band power shows the highest contribution to the model (100%), followed by Theta (59.52%), Alpha (54.02%), Gamma (46.92%) and Delta (32.79%) bands, as well as the resting state (25.80%) variable. Although with a smaller contribution, channels 104 (15.14%), 105 (8.39%), 103 (7.84%) and 26 (7.17%) complete the rank.

**Figure 11:**
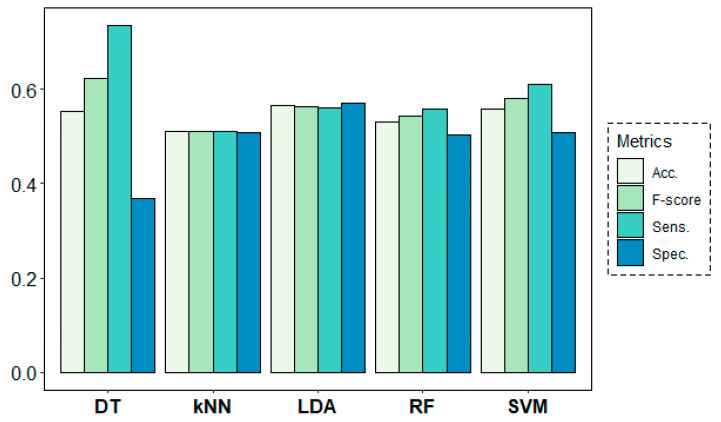
Classification results for the resting-state target variable.

Figure 11 shows the results of the classification models for the second research question. In this case, all models performed poorly, yielding only slightly better results than a random model in their classification task. Best accuracy results were obtained by LDA (56.46%), closely followed by SVM (55.88%) and DT (55.24%), not being enough to accept prediction ability for the different rs conditions with the given EEG-based predictors.

**Figure 12:**
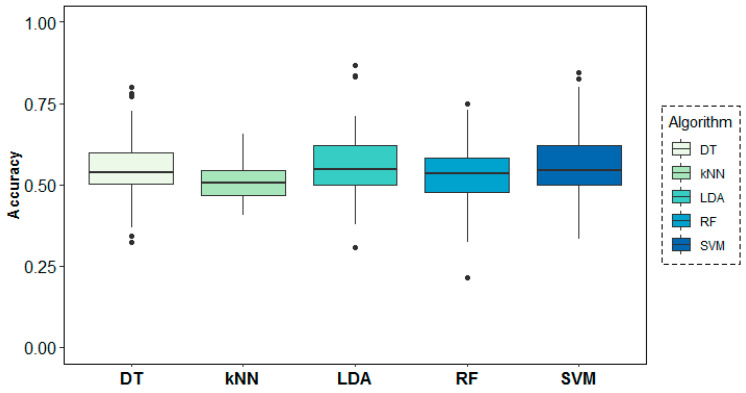
Classification accuracy results across folds (subjects) for the resting-state target variable.

Figure 12 shows the behaviour of the models above across folds. As expected, all accuracy results were found to be very stable across folds at around 50%. This reaffirms the difficulty to accept that the EEG data under analysis in this work allows to detect pre- and post-memory task states.

**Figure 13:**
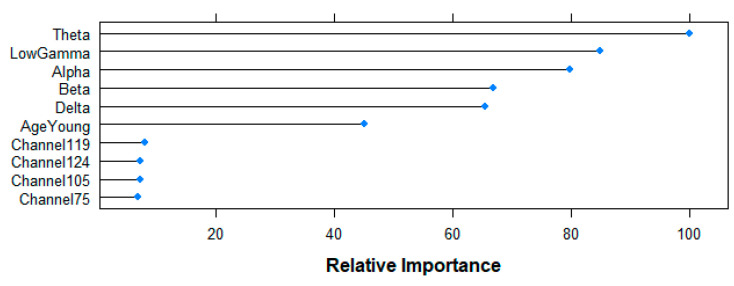
Relative importance of the top 10 features for the resting-state prediction task following RF-based Mean Decrease in Accuracy criteria.

Figure 13 shows the results of the top-10 variables used by the RF algorithm for its predictions. Relative importance of all frequency-bands is over 60% (Theta 100%, Gamma 84.85%, Alpha 79.83%, Beta 66.89%, Delta 65.39%), followed by the age-variable (45.15%), and channels 119 (8.04%), 124 (7.28%), 105 (7.17%) and 75 (6.81%) at a lower level. Having six variables with a relatively high importance in the model agrees with the poor performance observed for all the five models aiming at answering the second research question, since it suggests that the algorithm does not find one (or a few) important variable(s) that explain the difference between the two groups.

## 4. Discussion

### 4.1. Age-Related Modulations of Resting State Alpha Power after a Memory Task

Time domain results point to age-related differences between rs1 and rs2 in frontal and occipital electrodes in the Alpha band. Specifically, older participants showed reduced Alpha power as compared to their younger counterparts. Additionally, the later exhibited a significant increase in Alpha power from rs1 to rs2, whereas the former showed a more invariant pattern across resting state scans. Both MTW and STFT time-frequency analyses support these pattern of results in more anterior electrodes for certain time windows (mostly during the 120–150 s time-window). Converging evidence suggests age-related changes in posterior Alpha rhythm as the most characteristic phenomena that occur with brain aging. They include Alpha rhythm slowing [45,46,47], reduction of power [48], and a shift of sources in the posterior-to anterior direction [49]. The present results support power reductions in Alpha frequency with age during rest. Such reductions are prominent before and after task performance, reflecting small changes in resting state following a cognitive task. However, we observe a different pattern of results for young adults, showing increased Alpha power not only with respect to their older counterparts, but also after task performance. These findings suggest that resting state can be used as a predicting tool to assess changes in neural activity after task performance. What is more, resting state changes following task performance are more prominent in young compared to older adults, emphasising resting state modulations by age. The more invariant pattern of results observed in older compared to young participants may be related to a less evident neuroplasticity in the aging brain after such a short period of practice. A plausible explanation might be that older individuals need longer periods of cognitive practice in order to show a functional reorganization of neural networks [2].

The current results partially support a shift from posterior towards anterior neural sources that occurs. However, further analyses focused on source reconstruction should be conducted in order to elucidate which specific neural sources change with age in the resting brain following a cognitive task.

### 4.2. Validation of Morlet Wavelet Transform (MWT) and Short-Term Fourier Transform (STFT) Time-Frequency Methods with Resting-State Electroencephalogram (rs-EEG) Data

Regarding the results obtained for the time-frequency analysis, relevant statistical differences were obtained around the 140 s using both time-frequency analysis techniques. Smaller windows allowed the moment of change to be pin-pointed with higher accuracy, although they generally yielded a fewer amount of statistically relevant results due to the correction of the Type 2 error. Even if the statistical analysis showed similar results for both techniques, the computational complexity of the STFT and the MWT are not comparable. The most computationally costly operation in both techniques is the execution of the discrete Fourier transform (DFT) or the inverse discrete Fourier transform (IDFT). The asymptotical complexity of these operations for an input signal of size N is $O\left(N^{2}\right)$, although this complexity can be drastically reduced if the Cooley-Tukey implementation of the DFT/IDFT is applied [50]. This premise is only valid if the data segments where the DFT/IDFT are applied have a length of $2^{M};\;M\in \mathbb{Z}$, as is the case of the time-frequency analysis techniques considered in this research. In this case, the FT is termed fast Fourier transform (FFT) and its asymptotical complexity is reduced to O(NlogN). Considering a constant window length with no overlapping, the asymptotic complexity of the STFT scales linearly with the size of the input data vector (O(N)), whereas a complexity term of O(NlogN) is attained for the MWT. A qualitative analysis of the results shows little differences between both time-frequency analysis techniques, even if their computational cost is remarkably different. Hence, we can conclude that the STFT is better suited to perform time-frequency analysis on big scale rs-EEGs in terms of performance vs complexity.

### 4.3. Machine Learning-Based Models Successfully Predict Age Group in rs-EEG Data

Machine learning-based models allowed to answer two research questions regarding the use of EEG signals to classify subjects into young and older adults and into pre- and post-memory task conditions. For the first case, all models showed prediction accuracies over 68% and F-scores over 0.7, suggesting the promising ability of EEG signals to distinguish between the two age groups. Although there were no remarkable differences of performance between the tested algorithms, the DT and SVM-based models showed the best results, being the latter the one with the highest specificity. The analysis across-folds also showed the best accuracies to be achieved with a RF, where subjects were completely misclassified in very isolated occasions. The importance of variables in this prediction task showed power from the beta band to be the most discriminative feature. This differs from our previous results on the statistical analysis in the frequency domain where the Alpha band was found to show statistically significant differences between young and old groups of subjects. Nonetheless, differences between the main goals of the two analyses must also be taken into account. Whereas the former aimed at searching significant interactions between age and resting state variables, the latter only aimed at detecting the age-groups to which the subjects under study belonged using their EEG data. In addition, the technique used to rank the features was based on a RF, which is a non-linear algorithm. This may have caused non-linear relationships and patterns between variables to be discovered and emerge from the model, in contrast to what classic statistical analysis techniques are able to do. Therefore, further analysis using machine learning techniques in all frequency bands, but importantly in the Beta band, would be required to reaffirm our results and increase our knowledge and understanding about the underlying patterns followed by the human brain at aging.

The predictive models for the second research question suggested a difficulty classifying participants into the two resting states using their EEG patterns. None of the algorithms reached evident over-chance accuracy values. The analysis across folds followed the same patterns, showing almost random classification results in the study. The computation of the relative importance of the predictors suggested power from the Theta frequency band to be the principal contributor to the prediction. Nevertheless, the contribution was fairly shared among variables, suggesting no single or reduced group of good explanatory variables for the different resting state conditions. One could easily expect this pattern of results taking into account that the short duration of the cognitive task (half an hour) may not be enough to produce sustained changes in the functional organization of the brain or at least not significant enough to be detected by machine learning algorithms. The authors are currently working on further machine learning analyses, including resting state scans after the performance of an eight-week training period. We believe that this in turn will allow to perform a more reliable ranking of the EEG features.

### 4.4. General Conclusion

In this study, we provided evidence that resting state can be understood as a neural fingerprint of cognitive capacities and age-related changes in the functional reorganization of the brain after cognitive practice, resulting from a memory task. Besides the well-known reductions in Alpha power in older individuals, we were able to demonstrate the presence of differential brain activation patterns at rest in young and older individuals following a memory task. In this regard, older participants showed a more invariant resting state pattern across scans as compared to their younger counterparts, reflecting a less evident functional reorganization of neural networks following task performance, at least, in the context of the present short-duration cognitive task. In addition, our results were supportive of a posterior-towards-anterior shift in alpha power commonly observed in senior populations. Importantly, we were able to validate both MWT and STFT time-frequency methods and to conclude that the STFT is better suited to perform time-frequency analysis on big scale rs-EEGs in terms of performance vs. complexity. Last but not least, we employed different machine learning-based models that successfully allowed us to classify participants within their age group. Altogether, these findings greatly contribute to the development of multivariate tools for EEG data analysis and improve our understanding of age-related changes in the functional reorganization of the resting brain. Finally, these promising results in the context of a short-term cognitive task lay the ground for further analyses focused on the functional reorganization of resting state neural networks after long-term practice. In this regard, we plan to run similar analyses and compare young and older individuals before and after performing an 8-week training program. We expect both age and long-term practice effects on the functional organization of the resting brain.

## Figures and Tables

**Table 1:** Statistically significant results for the Alpha waveform per channel.

Channel Number	*p*-Value	F-Value	Channel Number	*p*-Value	F-Value
9	0.0160 *^,1^	6.1144	44	0.0024 **	9.9600
10	0.0167 *	6.0378	45	0.0003 ***	14.8917
11	0.0008 ***	12.2638	57	0.0148 *	6.2753
12	0.0048 **	8.5276	62	0.0107 *	6.9112
13	0.0007 ***	12.7786	67	0.0014 **	11.1674
14	3.81e-05 ****	19.5465	68	0.0108 *	6.8857
15	0.0028 **	9.6454	69	0.0063 **	7.9804
23	0.0091 **	7.2343	73	0.0069 **	7.7731
24	0.0026 **	9.7453	74	0.0140 *	6.3709
26	0.0036 **	9.1346	77	0.0083 **	7.4056
27	4.42e-05 ****	19.1796	79	0.0083 **	7.4139
28	0.0022 **	10.1651	82	0.0080 **	7.4865
29	0.0096 **	7.1297	84	0.0107 *	6.9041
31	0.0029 **	9.6025	88	0.0093 **	7.1879
36	0.0155 *	6.1841	90	0.0031 **	9.4123
37	0.0104 *	6.9625	91	0.0067 **	7.8477
38	0.0023 **	10.0323	95	0.0109 *	6.8636
39	0.0010 **	11.8744	96	0.0011 **	11.6654
40	0.0015 **	10.9990	99	0.0122 *	6.6548
41	4.99e-05 ****	18.8821	100	0.0150 *	6.2400
43	0.0145 *	6.3094	108	0.0148 *	6.2647

^1^ Statistically significant differences between groups in resting states after false discovery rate (FDR) correction (*: adjusted-*p* < 0.05, **: adjusted-*p* < 0.01, ***: adjusted-*p* < 0.001, ****: adjusted-*p* < 0.0001).
